# Reduced rate of copy number aberrations in mucinous colorectal carcinoma

**DOI:** 10.18632/oncotarget.4706

**Published:** 2015-07-25

**Authors:** Niek Hugen, Femke Simmer, Leonie J.M. Mekenkamp, Miriam Koopman, Evert van den Broek, Johannes H.W. de Wilt, Cornelis J.A. Punt, Bauke Ylstra, Gerrit A. Meijer, Iris D. Nagtegaal

**Affiliations:** ^1^ Department of Surgery, Radboud university medical center, 6500 HB Nijmegen, The Netherlands; ^2^ Department of Pathology, Radboud university medical center, 6500 HB Nijmegen, The Netherlands; ^3^ Department of Internal Medicine, Medical Spectrum Twente Enschede, 7500 KA Enschede, The Netherlands; ^4^ Department of Medical Oncology, University Medical Center Utrecht, 3508 GA Utrecht, The Netherlands; ^5^ Department of Pathology, VU University Medical Centre, 1007 MB Amsterdam, The Netherlands; ^6^ Department of Medical Oncology, Academic Medical Center University of Amsterdam, 1100 DD Amsterdam, The Netherlands

**Keywords:** colorectal adenocarcinoma, mucinous carcinoma, comparative genomic hybridization, copy number profile

## Abstract

**Background:**

Mucinous carcinoma (MC) is found in 10%–15% of colorectal cancer (CRC) patients. It differs from the common adenocarcinoma (AC) in histopathological appearance and clinical behavior.

**Methods:**

Genome-wide DNA copy number and survival data from MC and AC primary CRC samples from patients from two phase III trials (CAIRO and CAIRO2) was compared. Chromosomal copy number data from The Cancer Genome Atlas (TCGA) was used for validation. Altogether, 470 ACs were compared to 57 MCs.

**Results:**

MC showed a reduced amount of copy number aberrations (CNAs) compared with AC for the CAIRO/CAIRO2 cohort, with a median amount of CNAs that was 1.5-fold lower (*P* = 0.002). Data from TCGA also showed a reduced amount of CNAs for MC. MC samples in both cohorts displayed less gain at chromosome 20q and less loss of chromosome 18p. A high rate of chromosomal instability was a strong negative prognostic marker for survival in MC patients from the CAIRO cohorts (hazard ratio 15.60, 95% CI 3.24–75.05).

**Conclusions:**

Results from this study indicate that the distinct MC phenotype is accompanied by a different genetic basis when compared with AC and show a strong association between the rate of chromosomal instability and survival in MC patients.

## INTRODUCTION

Colorectal cancer (CRC) is categorized by histological subtype according to the WHO classification. The majority of patients (∼85%) is diagnosed with an adenocarcinoma not otherwise specified (AC). Mucinous adenocarcinoma (MC) is detected in 10%-15% of patients and is characterized by abundant extracellular mucin lakes that comprise more than half of the tumor volume. [1] MC differs from AC in both clinical and pathological presentation. [2, 3] MC is more frequently found in the proximal colon and at a higher stage at presentation than AC. [2, 3] Also, the response to therapies varies between MC and AC as patients with MC show a poorer response to palliative chemotherapy compared with AC, resulting in a worse survival. [4–7] These findings suggest a distinct genetic background of MC.

There are two major pathways through which genomic instability can occur in CRC, namely chromosomal instability (CIN) and microsatellite instability (MSI). CIN is found in the majority (∼85%) of CRCs and is a type of genetic instability in which chromosomal aberrations accumulate, leading to an altered expression of tumor suppressor genes and oncogenes. [8] MSI accounts for the remaining 15% of CRCs and is caused by a defective DNA mismatch repair mechanism that leads to a clonal change in the number of microsatellites. [9] Tumors with MSI are more commonly found in MC patients compared with AC patients, and exhibit less (CNAs). [5, 10]

Although MSI is more common in MC than in AC, the vast majority of MCs supposedly still develops through the CIN pathway. Unfortunately, most large-scale genomic studies of CRC did not address differences between histological subtypes and focused mainly on AC. Specific DNA CNAs that cause gene dosage effects in oncogenes and tumor suppressor genes, typically occur during adenoma to carcinoma progression, and thus are an integral part of the pathogenesis of CRC. [11] Therefore, analysis of DNA CNAs between MC and AC in a sufficiently large collection of samples may generate more insight into an early and possibly diverging event in cancer development.

In this study we use DNA copy number data from primary tumor samples of patients with MC or AC who participated in two phase III clinical trials. These data were used to test whether the distinct MC and AC phenotypes relate to differences in genomic profiles. Molecular characterization of MC may improve our understanding of the reduced response rate to systemic therapies and therefore contributes to the development of targeted treatment modalities for MC.

## MATERIALS AND METHODS

### Patients and materials

For this study we used clinical and genome data of patients from two randomized controlled trials and validated our findings with data from TCGA.

### CAIRO and CAIRO2 cohorts

We used high resolution array comparative genomic hybridization (aCGH) data that were generated from DNA isolated from formalin-fixed and paraffin-embedded (FFPE) primary tumors, which was hybridized against paired germ-line DNA samples. The processes of sample selection, DNA isolation and aCGH data have been described previously. [12] Samples were derived from patients who participated in the CAIRO study (CKTO 2002–07, ClinTrials.gov; NCT00312000) [13] or CAIRO2 study (CKTO 2005–02, ClinTrials.gov; NCT00208546) [14] of the Dutch Colorectal Cancer Group (DCCG). These phase III trials had different systemic regimens as first-line treatment for CRC patients with metastatic disease. In the CAIRO study, patients were randomly assigned to either sequential or combination treatment with capecitabine and irinotecan, followed by oxaliplatin. In the CAIRO2 study patients were randomized between treatment with capecitabine, oxaliplatin and bevacizumab, with or without the addition of cetuximab. All patients had given written informed consent prior to study entry, which also included translational research on tumor tissue. aCGH was performed on a subset of the patients from these trials. [12] In the CAIRO2 study the aCGH was only performed on material from the control arm since the addition of cetuximab in the experimental arm yielded a worse outcome. Furthermore, only tumors with paired germ-line tissue, and with areas of high tumor cell percentage available (>70%) had been included. A total of 349 high quality DNA copy number profiles were generated. Tumors were classified according to the guidelines of the World Health Organization. If more than 50% of the tumor consisted of extracellular mucin it was classified as MC. [1] AC was defined as a tumor without extracellular mucin. Only tumors that were categorized as MC or AC were included in our analyses. Tumors with microsatellite instability (MSI) usually exhibit limited CNAs, and therefore form a separate entity among CRCs. These patients (*N* = 31) were excluded from the present study. In the current study DNA copy number profiles of 17 MC and 135 AC patients from the CAIRO study and 12 MC and 100 AC patients from the CAIRO2 study were compared.

### The cancer genome atlas cohort

To validate findings from the CAIRO cohorts, copy number information for MC and AC samples from The Cancer Genome Atlas (TCGA) data portal was analyzed. On 27 January 2014 all available colon adenocarcinoma level 3 copy number data were downloaded from the TCGA Data Portal using the Data Matrix (https://tcga-data.nci.nih.gov/tcga/dataAccessMatrix.htm). TCGA copy number data had been generated with Affymetrix SNP 6.0 arrays (Santa Clara, USA). Only data obtained from primary tumors was used. The histopathological designation as provided by TCGA was used, and only tumors that were categorized as MC or AC were selected. Furthermore, MSI tumors were excluded from the analyses. In total, DNA copy number profiles of 28 MC and 235 AC patients were compared.

### Clinicopathological data

For each patient, the following clinicopathological characteristics were available: age, gender, site of primary tumor, number of metastatic sites involved, invasion depth, lymph node status, MSI status and histological subtype. Tumors from the TCGA cohort were classified as proximal if they were found in the cecum, ascending colon or transverse colon, up to the splenic flexure, and were classified as distal if they were found in the descending colon or sigmoid colon. MSI status was determined by immunohistochemistry with antibodies against MLH1, MSH2, MSH6 and PMS2. MSI analysis was performed on indication by PCR followed by GeneScan analysis for MSI markers (BAT25, BAT26, BAT40, D2S123, D5S346, D17S250). [15, 16] Differences in baseline characteristics between groups were determined using Fisher's exact testing. Statistical analyses were two-sided and *p*-values < 0.05 were considered significant.

### Processing of aCGH and SNP array data

Array CGH was performed using customized Agilent oligonucleotide arrays. Methods of DNA extraction, labeling, hybridization and scanning were previously described [17] and the exact array design can be found online in the Gene Expression Omnibus (GEO) (GPL8687 http://www.ncbi.nlm.nih.gov/geo). The probes were mapped to human reference sequence GRCh37/hg19 (February 2009). The statistical programming language R was used for data processing. The quality of the aCGH DNA copy number profiles was assessed by calculating the median absolute deviation (MAD) from the log2ratios of signal intensities from tumor- and paired germ-line DNA. A MAD value of 0.4 and smaller was used as a quality criterion, which all DNA copy number profiles passed. A wave-smoothing algorithm was applied on the profiles and the profiles were median normalized and corrected for tumor cell percentage using the R package ‘CGHcall’. [18] For segmentation the R package ‘DNAcopy’ was used. [19] Next, mode normalization was performed. Subsequently, the DNA copy number (deletion, loss, neutral, gain, or amplification) was determined for each segment using the R package ‘CGHcall’. [12] This data was used to generate genome-wide frequency plots and box plots with the number of aberrations. For further analyses the dimensions of the aCGH data set were reduced using the R package ‘CGHregions’ (averror = 0.01). [20] This step reduced the calls into subregions. Each subregion consisted of a series of neighboring clones on the chromosome whose aCGH-signature was shared by all clones. With this step 2010 subregions were obtained with a median size of 0.5 Mb (interdecile range = 110 kb–2 Mb).

The TCGA level 3 SNP6 data consisted of copy number values (log2ratios) generated with ‘nocnv’ segmentation. For the genome-wide frequency plots, information was extracted from the downloaded files with LINUX shell and BEDTools. [21] By extracting the genomic positions of all segment ends, a file with unique genomic positions was made. This gave 52654 genomic positions distributed over all chromosomes. Next, for each TCGA sample the log2 ratio at these positions was collected. In R these copy number values were converted into calls. The threshold was set as previously described. [22] Values lower than −0.23 were assigned copy number loss, and values higher than 0.2 were assigned gain, all other values were assigned neutral. These values correspond to 30% of the tumor cells with that CNA. This data was used to generate the frequency plots using functions of the R package ‘CGHbase’.

### Analysis of the level of chromosomal instability

For each sample we counted the number of probes called as loss, neutral or gain and subsequently calculated the percentage of probes with an aberrant call. The distribution of this level of chromosomal instability was plotted in box-plots. To assess whether the distribution of MC and AC samples was different, the Wilcoxon rank sum test was used (also known as Mann-Whitney test).

### Identification of regions with differential copy number

DNA copy number information of the 2010 subregions was analyzed in a supervised way. MCs from both CAIRO studies were compared with ACs from the same studies. Per sample group, the frequencies of losses, neutrals and gains were determined for each region. To calculate the statistical significance of DNA copy number differences between MC and AC the Wilcoxon rank sum test was used, and a correction for multiple testing was performed with the Benjamini-Hochberg procedure. An adjusted *p*-value < 0.01 was considered statistically significant. The TCGA data were used for validation of the differential subregions identified with the CAIRO/CAIRO2 samples. Frequency plots were generated for subregions that showed differences in copy number and a Wilcoxon rank sum test was performed on the calls to determine significant DNA copy number differences between MC and AC, followed by correction for multiple testing with the Benjamini-Hochberg procedure.

### Survival analysis

To determine the impact of CIN on survival in MC and AC patients, groups were divided into CIN high and CIN low. The threshold was set at the median level of CIN of all samples of the CAIRO cohorts which was 29.66%. Patients who demonstrated a CIN rate below the median were considered CIN low and consequently, patients who demonstrated a CIN rate that was above the median, were considered CIN high. Overall survival (OS) was defined as the interval between the date of randomization until the date of death of any cause or until last follow-up. Patients who were alive at the end of follow-up were censored in the survival analyses. OS curves were estimated using the Kaplan–Meier method and compared with the log-rank test. Multivariable analysis of OS was performed using the Cox proportional hazard model. Statistical analyses were performed with the statistical software package SPSS 20.0 (SPSS Inc, Chicago, Illinois, USA).

## RESULTS

### Clinicopathological data of the CAIRO, CAIRO2 and TCGA cohort

The baseline characteristics on MC and AC patients from the CAIRO/CAIRO2 cohort are presented in Supplementary Table S1. MC patients were more commonly over 60 years of age than AC patients (86.3% versus 62.5%, *P* = 0.04). There were no other significant differences in clinicopathological characteristics between MC and AC patients. Data on survival between AC and MC in advanced stage disease were published previously on these series. [5] Clinicopathological data on colon cancer patients from the TCGA cohort is presented in Supplementary Table S2. In MC patients from the TCGA cohort, tumors were more commonly located in the proximal colon than in AC patients (78.6% versus 49.4%, *P* = 0.004). The distribution of tumors was not different in the CAIRO/CAIRO2 cohort. There were no further substantial differences in baseline characteristics.

### Different copy number profiles between MC and AC

**Figure 1:**
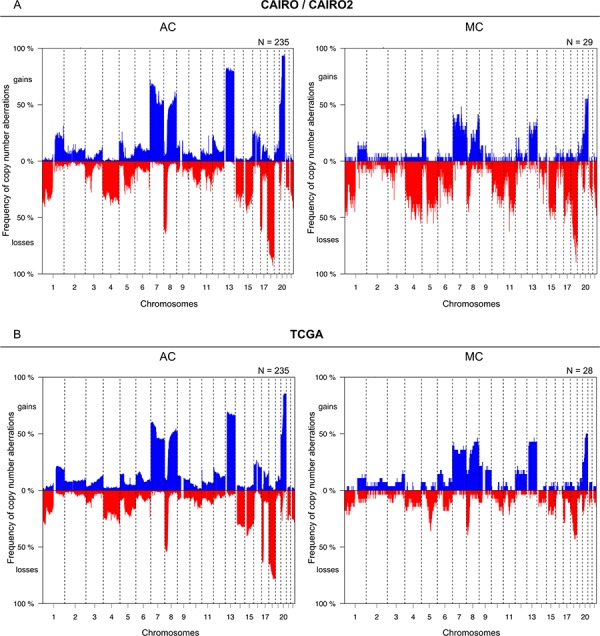
Comparison of the overall frequencies of DNA CNAs between MC and AC Genome-wide frequency plots of DNA CNAs detected in the CAIRO/CAIRO2 **A.** and TCGA **B.** cohorts. The x-axis displays clones on the array ordered by chromosomal map positions of the clones. The y-axis displays the percentage of tumors with gains (above zero; blue) or losses (below zero; red). Boundaries of chromosomes are indicated by dotted lines.

**Figure 2:**
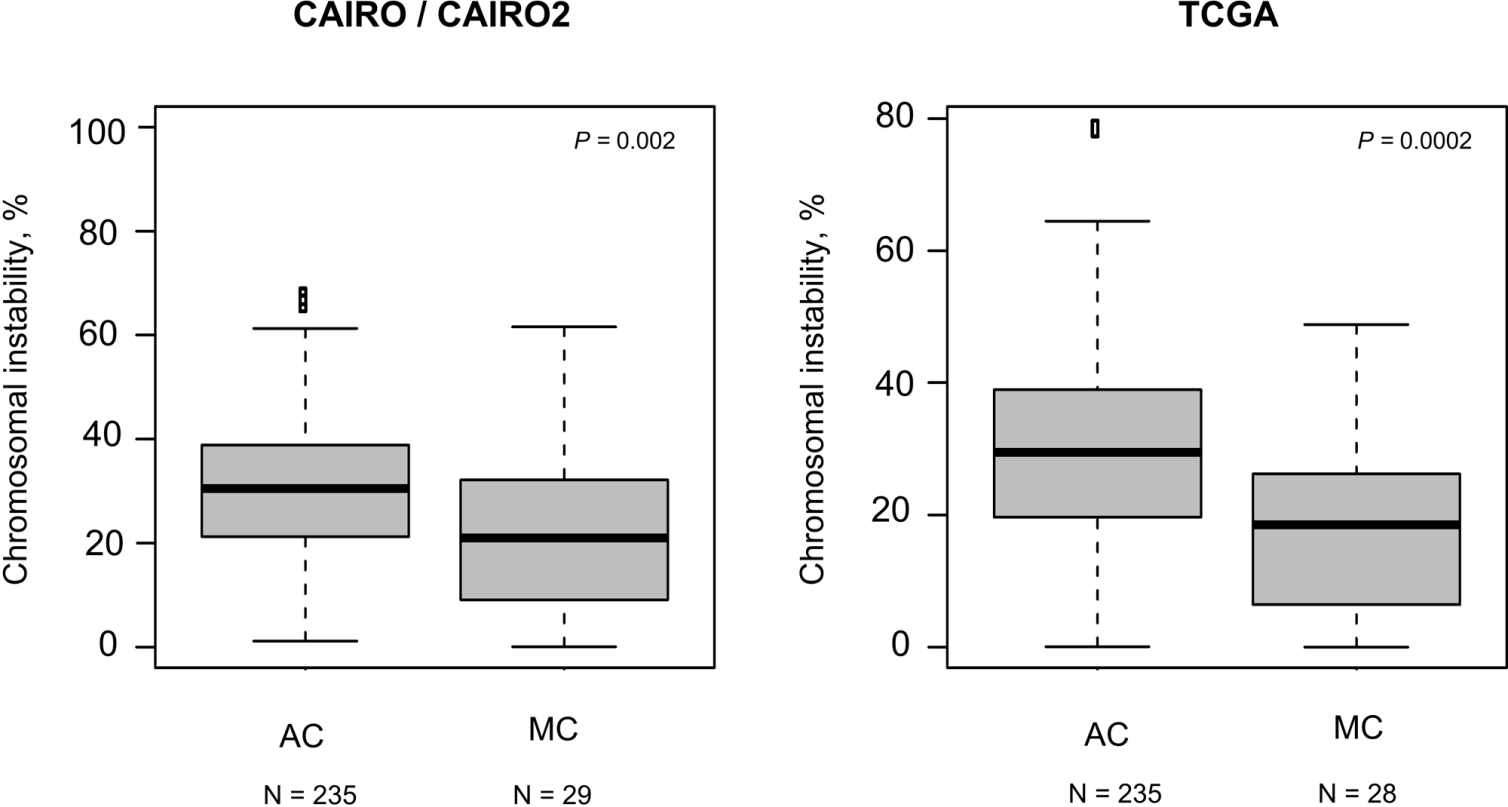
Levels of CIN For each sample the percentage of probes with an aberrant call was calculated. The box plots show per cohort the distribution of the percentage of probes with an aberrant call for the AC and MC samples. In both cohorts a lower median chromosomal instability for MC is observed.

The frequency of CNAs in MC and AC patients from the CAIRO/CAIRO2 and TCGA cohorts are depicted in Figure 1. These genome-wide profiles of CNAs of MC and AC patients appeared rather similar in both cohorts, but overall MCs displayed a lower level of chromosomal instability (Figure 2, left). In the MCs from the CAIRO/CAIRO2 cohort a median of 21% of the genome showed either deletions, losses, gains or amplifications, compared with 31% for the ACs, *P* = 0.002. For the TCGA cohort this was 19% for MC versus 29% for AC, respectively (*P* = 0.0002). In the CAIRO/CAIRO2 cohort there were particularly differences in the overall frequencies of the gains between AC and MC (Supplementary figure S1).

**Figure 3:**
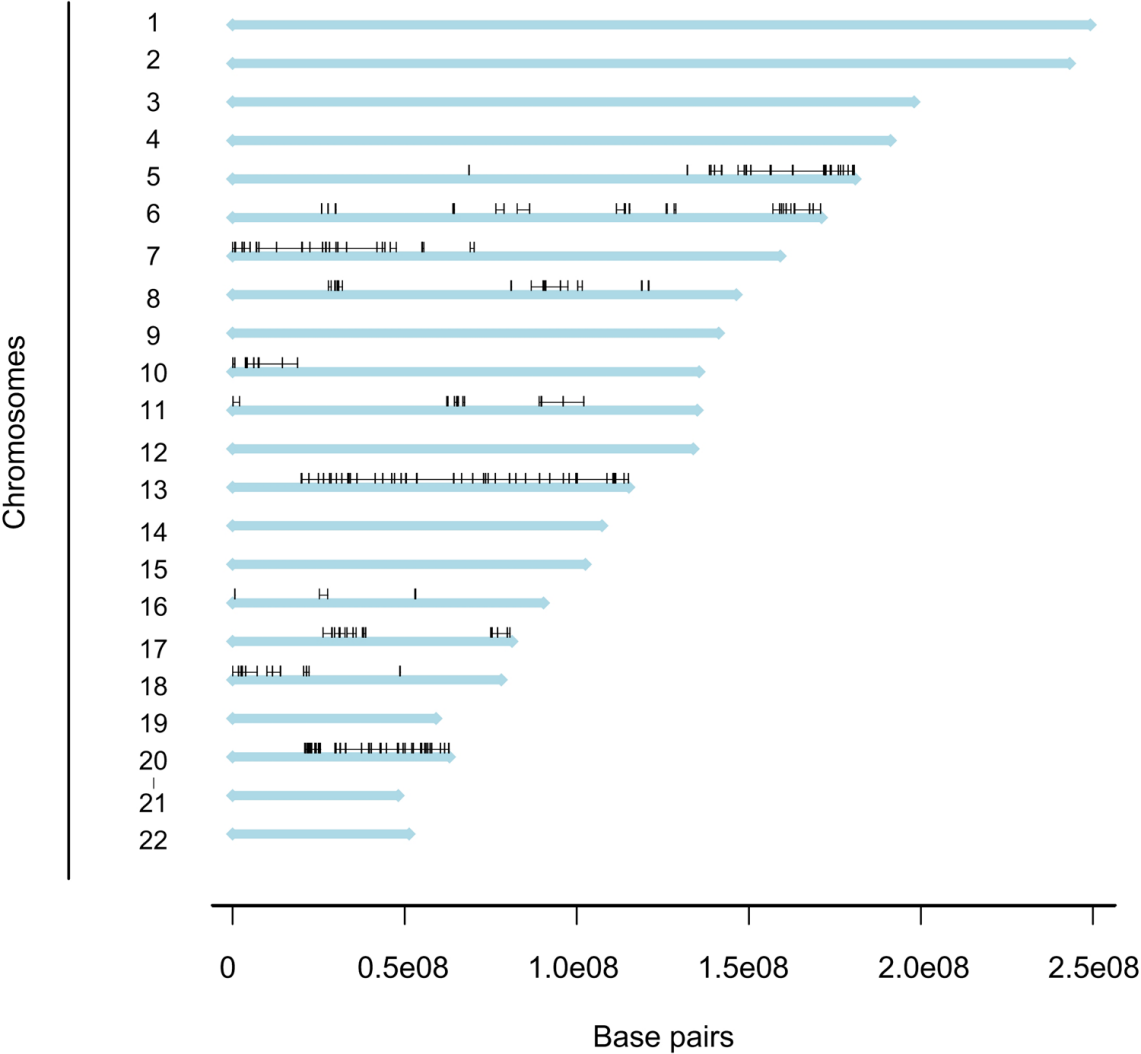
Distribution of differentiated regions Genome plot depicting a linear view of the chromosomes and distribution of the regions with a significant differential DNA copy number frequency between MC and AC patients in the CAIRO/CAIRO2 cohort. The 234 regions are localized on 11 chromosomes.

Next, analyses were performed to further identify chromosomal subregions with significant differential copy number. We identified 234 significantly differential subregions in the CAIRO/CAIRO2 cohort (*P* = 0.01; Figure 3). These subregions were located on chromosomes 5, 6, 7, 8, 10, 11, 13, 16, 17, 18 and 20.

### Validation in TCGA data

**Figure 4:**
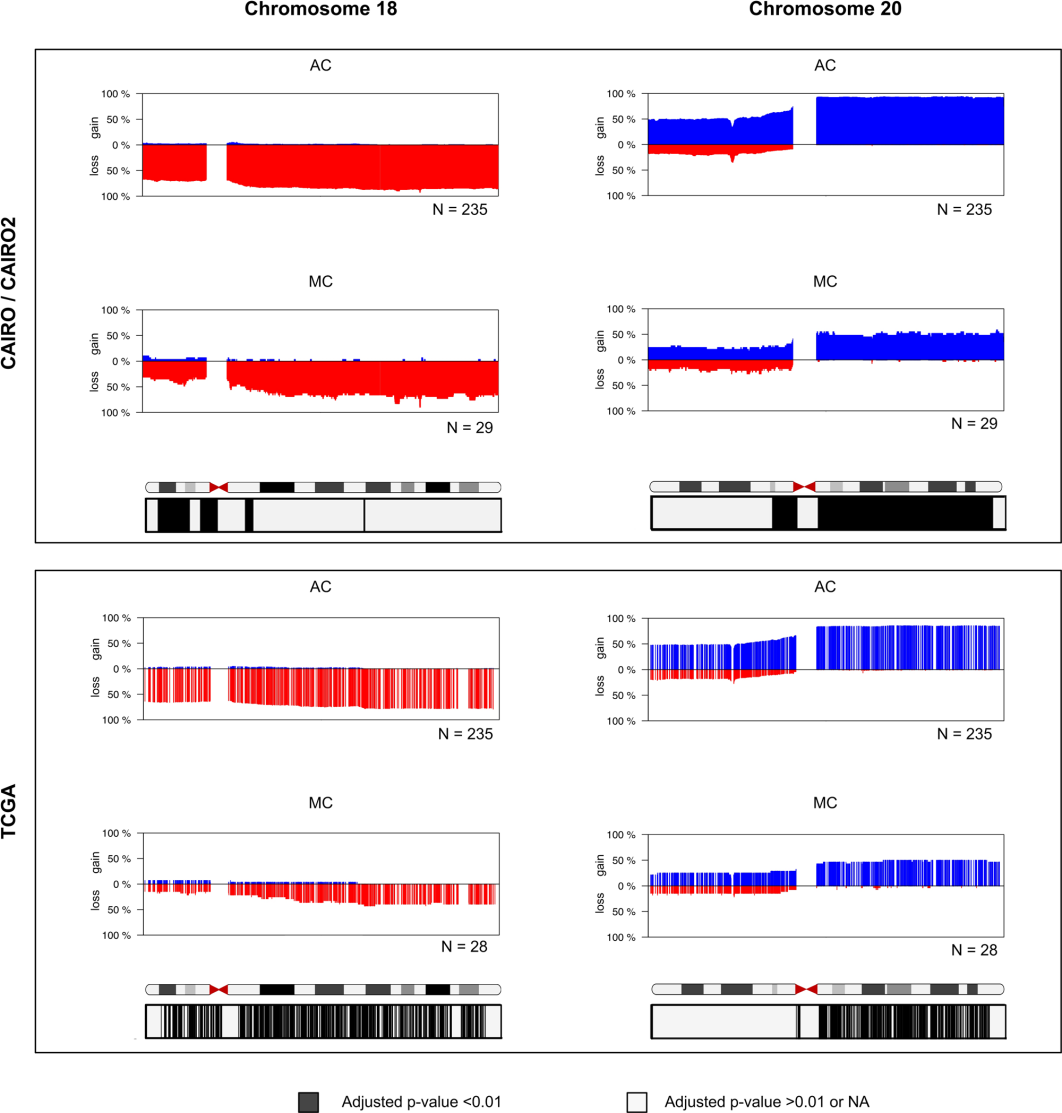
Frequency plots of DNA CNAs in chromosome 18 and chromosome 20 Detailed view of chromosomes with the differential regions of the CAIRO/CAIRO2 cohort that are validated in the TCGA data set. The y-axis displays the percentage of tumors with gain (above zero; blue) or los (below zero; red). The chromosomes represent ideograms with chromosomal bands. The corrected *p*-values obtained with statistical significance testing and correction for multiple testing are depicted in boxes below the plots. Black represents adjusted *P* < 0.01 and indicated a significant difference in DNA copy number between AC and MC; grey represents adjusted *P* > 0.01 and no indication of a significant difference. The significant loci obtained from the CAIRO/CAIRO2 cohort on 18p and 20q can be merged into two regions of main interest: chr18:122131–13971462 and chr20:29833609–62880524. For the region of interest on p18 34% of MC patients showed a loss in the CAIRO/CAIRO2 cohort, compared with a loss in 69% in AC patients. For the region of interest on q20 52% of MC patients in the CAIRO/CAIRO2 cohort showed a gain, while AC patients showed 93% gain or amplification.

To confirm the findings from the CAIRO/CAIRO2 cohort, data from TCGA were analyzed. The TCGA data not only represents another patient cohort, but also a different methods to determine DNA CNAs, since single channel SNP arrays were used, rather than CGH arrays. In addition, no paired normal DNA was used and DNA was isolated from fresh frozen material. MC samples displayed a reduced rate of CNAs than AC samples (Figure 2, right). Furthermore, MC samples in this cohort showed a significantly differential copy number for chromosome 18 and 20 (Figure 4), but not for the other chromosomes with significant differential subregions for the CAIRO/CAIRO2 cohort (chromosomes 5, 6, 7, 8, 10, 11, 13, 16 and 17). This included 13q gain, which showed a highly significant difference between AC and MC in the CAIRO/CAIRO2 cohort, while in the TCGA cohort this could not be confirmed (Supplementary Figure S2).

### Different copy numbers at chromosome 18 and 20

MC patients displayed significantly less losses at chromosome 18 compared with AC patients in both cohorts. In the TCGA cohort, this comprised nearly the entire chromosome, but for the CAIRO/CAIRO2 cohort, this was mainly restricted to regions of the p-arm (Figure 4). The significant loci on 18p for the CAIRO/CAIRO2 cohort were merged into one region of main interest of 14 Mb, which involved almost the entire p-arm (18p11.32–18p11.21). For this region of interest, 34% of MC patients showed a loss in the CAIRO/CAIRO2 cohort, compared with 69% of AC patients.

In the TCGA data these percentages were 14% and 64% respectively. At the q-arm of chromosome 20 both the CAIRO/CAIRO2 and TCGA cohorts showed less gains in MC patients compared with AC patients (Figure 4). This region of interest at chromosome 20 was 33 Mb and comprised essentially the entire q-arm (20q11.21–20q13.33). A gain at this region was found in 52% MC patients in the CAIRO/CAIRO2 cohort, while AC patients showed 93% gain or amplification. The percentages of these gains were 46% and 84%, respectively for the TCGA data.

### Survival is related to CIN status in MC patients

**Figure 5:**
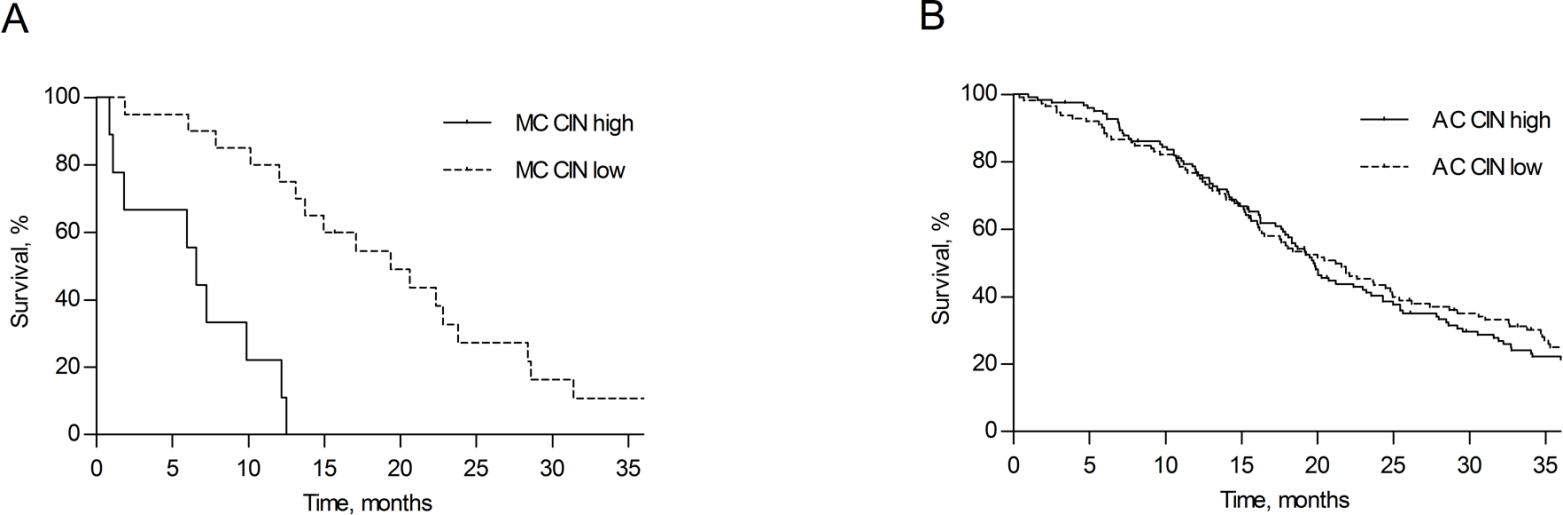
Survival according to rate of chromosomal instability Overall survival in MC **A.** and AC **B.** colorectal cancer patients from the CAIRO/CAIRO2 cohort, distributed according to a high or a low rate of chromosomal instability (CIN).

The relation between survival and either a high or low rate of CIN was explored. There were 112 AC and 20 MC patients in the CIN low group versus 123 AC and 9 MC patients in the CIN high group. OS rates in MC patients were dependent on the rate of chromosomal instability. MC CIN high patient had a statistically significant poorer OS compared with MC CIN low patients. MC CIN high patients had a median OS of 6.6 months (95% CI 4.8–8.4) versus 19.4 months (95% CI 11.7–27.0) for MC CIN low patients (Figure 5a). An OS difference according to CIN status was not observed in AC patients, with a median OS of 19.7 months (95% CI 17.8–21.7) for AC CIN high patients compared with 21.2 months (95% CI 16.3–26.1) in AC CIN low patients (Figure 5b). Interestingly, MC CIN low patients had an OS that was comparable to that of AC patients. Also, in the multivariable Cox regression analysis a high rate of CIN was a strong negative prognostic marker for OS in MC patients from the CAIRO cohorts with a hazard ratio of 15.60 (95% CI 3.24–75.05, Supplementary Table S3).

## DISCUSSION

MC is considered a unique subtype of CRC based on its histopathological appearance and clinical behavior. This study investigated whether the distinct visual microscopic pathological characteristics of MC are associated with different genetic aberrations when compared with AC in MSS primary tumors.

In CRC, CIN and MSI are two well-defined genomic pathways that are involved in carcinogenesis. MSI is found in approximately 15% of CRCs, but is more commonly found in MCs than in ACs. [5, 9, 10, 23] Although MSI and CIN are not mutually exclusive, CNAs are far less common in MSI than MSS tumors. [24, 25] In the present series of MSS CRCs an overall lower level of genetic instability was observed in MC compared with AC. As to specific DNA CNAs, the frequency of gain of chromosome 20q and the frequency of loss of chromosome 18p was significantly lower in MC. Chromosomal gains of 20q and losses of 18p are among the most common molecular aberrations in CIN induced CRCs and since their occurrence is associated with progression from adenoma to carcinoma they are considered early genetic events. [11, 26] It should therefore be noted that these regions are consequently most prone to reach statistical significance if there are overall differences in genetic instability as we observed for AC versus MC. Notwithstanding, these regions could be less contributory in MC development.

Amplification of 20q is an early molecular event and considered one of the key events that may induce the malignant process. [11, 27, 28] Gain of a chromosomal region at 20q is present in over 60% of CRCs. [25, 28, 29] There are several common regions of overlap of the highest level of gains at the 20q arm, which makes it plausible that multiple genes are involved in CRC development. The exact mechanism through which this occurs has not yet been elucidated, but an increasing number of genes that would be responsible for this 20q amplicon-driven progression has been identified. [27, 30, 31] Gain of 20q has also been associated with a poor prognosis in CRC patients. [32] Previously, we found that prognosis of MC patients in advanced stage disease was worse compared with AC patients, due to a decreased response to palliative chemotherapy. [5] Our group identified chromosomal regions that were associated with a decreased responsiveness to the addition of irinotecan in advanced colorectal cancer. [12] However, these specific regions did not differ between MC and AC patients in the current study. The poor response to chemotherapy may be related to the deviant pattern along which metastatic disease spreads in MC patients. Compared with AC, MC is less likely to present with liver metastases only, whereas intra-abdominal metastases are observed in more than half of all cases with advanced disease. [33] One study that analyzed CNAs between patients with different metastatic patterns showed that gain of the 20q chromosomal arm was associated with liver-specific metastases, suggesting a role in the process of liver metastasis in CRC. [34] Patients who did not develop metastatic disease and patients with peritoneal metastases showed a gain of 20q less frequently. [34, 35] These findings fit very well with the aberrant metastatic pattern that has been observed in MC patients.

Losses of chromosome 18q and 18p are seen in two third of CRC patients. [36] Especially loss of 18q is a well-known aberration that has been associated with adenoma to carcinoma progression. However, loss of 18p has also been found to be an early genetic change in primary CRC. [26, 37] It is unknown what genes on 18p can be held accountable for the malignant progression. Gain of chromosome 13q is found in approximately half of CRCs. [29, 36, 38] and is associated with adenoma to carcinoma progression as well. [37] Interestingly, in the present study we found less gain for virtually the entire 13q-arm in the CAIRO/CAIRO2 cohort, but this could not be confirmed with data from the TCGA cohort.

We found that a high level of CIN was associated with a poor outcome in MC patients, but not in AC patients. Previously, CIN has been associated with a poor prognosis, mostly in stage II and III CRC. [39] Apparently, this also accounts for MC stage IV patients. It has been suggested that abnormalities of the spindle checkpoint drive CIN. [40] Overexpression of *AURKA* (located on Chr 20q13, encoding the kinase Aurora-A) or loss of *CHEK2* (located on Chr 22q12, encoding the DNA damage checkpoint kinase Chk2) increase microtubule assembly, promoting CIN. [41] The mitotic checkpoint may thus provide a novel therapeutic target to improve overall survival and/or to modify response to chemotherapy. [42]

It is increasingly acknowledged that CRC is a heterogeneous disease and there is ample evidence that tumors differ on a molecular level. Previously, two studies that used allelotyping PCR for a few loci showed that lower allelic imbalance is associated with MC. [43, 44] In the current study, based on data from two independent cohorts we conclude that the distinct phenotype of MC is accompanied by a different genome-wide genetic profile when compared with AC, marked by a reduced rate of DNA CNAs overall, as well as less frequent gain of 20q and less frequent loss of 18p. Therefore, it may be possible that CIN plays a less prominent role in MC development. The differences in CNAs were found in a metastatic cohort (CAIRO/CAIRO2 cohort) as well as in a cohort with more early stage tumors (TCGA cohort), supporting the view that there is a genetic distinction between MC and AC that transcends the stage of presentation. Evidently, the consequences of chromosomal aberrations on gene expression levels eventually determine the functional phenotype. This study did not provide an insight into the specific pathway along which MCs developed, but was able to demonstrate that MC differed from AC on a molecular level. Since the DNA copy number effect was seen for large chromosomal regions and throughout the genome, the effects are probably due to genomic imbalance and alterations at multiple genes rather than specific genes. Further studies that can assess molecular differences between MC and AC with a higher resolution (e.g. next-generation sequencing) are therefore needed. Previously, it has been reported that mutation rates in the therapeutically important RAS/RAF/MAPK and PI3K/AKT pathways are significantly higher in MC than in AC. [5, 45–47] Moreover, MC is more frequently associated with MSI and the CpG island methylator (CIMP) phenotype. [45, 46, 48] Although these features are not exclusive for MC patients, they do suggest that MC may develop through an alternative genetic instability pathway than CIN, which may explain the distinct tumor behavior and response to therapies. Due to low number it was not possible to compare differences in copy number changes between MSI MC and MSS MC patients in this study. It will be important to further investigate the molecular background of MC to increase knowledge on tumor behavior and to explore opportunities for targeting therapies. These data will enable clinicians to improve prediction of the course of disease and response to systemic treatment.

## SUPPLEMENTARY TABLES AND FIGURES


