# Supplementary material for: Reduced rate of copy number aberrations in mucinous colorectal carcinoma
Source: Oncotarget. 2015 Jul 25;6(28):25715–25. doi: 10.18632/oncotarget.4706 (PMC4694861; doi:10.18632/oncotarget.4706)
Supplement: Supplementary file 1 [file oncotarget-06-25715-s001.pdf]

SUPPLEMENTARY TABLES AND FIGURES

Supplementary Table S1. Clinicopathological characteristics of metastatic CRC patients who were included in the CAIRO/CAIRO2 cohort

Features	CAIRO/CAIRO2				*p*-value
	AC		MC		
	235	(%)	29	(%)	
**Sex**					0.4
Female	93	39.6	9	69.0	
Male	142	60.4	20	31.0	
**Age at diagnosis**					0.04
Median (range)	64	(36–81)	67	(50–79)	
<45	9	3.8	0	0	
45–59	79	33.6	4	13.8	
60–74	126	53.6	19	65.5	
≥75	21	8.9	6	20.7	
**Location of primary**					0.3
Colon	120	51.1	15	51.7	
Rectum	71	30.2	6	20.7	
Rectosigmoid	33	14.0	8	27.6	
Multiple locations	1	0.4	0	0	
Unknown	10	4.3	0	0	
**Invasion depth**					0.9
T1–2	18	7.7	1	3.4	
T3	170	72.3	23	79.3	
T4	42	17.9	5	17.2	
Unknown	5	2.1	0	0	
**Lymph node status**					0.6
N0	65	27.7	9	31.0	
N1	74	31.5	12	41.4	
N2	82	34.9	7	24.1	
Unknown	14	6.0	1	3.4	
**Number of metastases**					0.4
1	123	52.3	12	41.4	
>1	111	47.2	17	58.6	
Unknown	1	0.4	0	0	

Fisher's exact test was applied.

Supplementary Table S2. Clinicopathological characteristics of patients from the TCGA cohort

Features	TCGA				
	AC		MC		*p*-value
	235	(%)	28	(%)	
**Sex**					0.4
Female	96	40.9	14	50.0	
Male	139	59.1	14	50.0	
**Age at diagnosis**					0.8
Median (range)	68	(31–90)	68	(45–90)	
<45	10	4.3	0	0.0	
45–59	53	22.6	7	25.0	
60–74	107	45.5	12	42.9	
≥75	65	27.7	9	32.1	
**Location of primary**					0.004
Proximal colon	116	49.4	22	78.6	
Distal colon	119	50.6	6	21.4	
**Invasion depth**					0.6
Tis	1	0.4	0	0.0	
T1	7	3.0	0	0.0	
T2	43	18.3	3	10.7	
T3	162	68.9	21	75.0	
T4	22	9.4	4	14.3	
**Lymph node status**					0.4
N0	131	55.7	13	46.4	
N1	58	24.7	6	21.4	
N2	45	19.1	9	32.1	
Unknown	1	0.4	0	0.0	
**Distant metastasis**					1.0
M0	176	74.9	21	75.0	
M1	36	15.3	4	14.3	
Mx	23	9.8	3	10.7	

Fisher's exact test was applied.
Proximal colon: from cecum up to the splenic flexure.
Distal colon: descending colon, sigmoid and rectosigmoid junction.

Supplementary Table S3. Multivariate analysis with 95% confidence interval (CI) on overall survival in MC patients from the CAIRO/CAIRO2 cohort

Features	HR	95% CI
**Sex**		
Male	1	
Female	0.67	0.18–2.51
**Age group**		
45–59	1	
60–74	0.26	0.05–1.47
≥ 75	0.47	0.06–3.52
**Tumor location**		
Colon	1	
Rectum	1.46	0.29–7.44
Rectosigmoid	0.99	0.18–5.25
**Invasion depth**		
T1–2	6.04	0.33–109.54
T3	1	
T4	2.47	0.50–12.18
**Lymph node status**		
N0	1	
N1	1.31	0.32–5.29
N2	1.31	0.30–5.68
Unknown	1.95	0.10–38.04
**Number of metastases**		
1	1	
> 1	2.14	0.56–8.13
**CAIRO study**		
CAIRO	1	
CAIRO2	0.79	0.27–2.28
**CIN status**		
CIN low	1	
CIN high	15.60	3.24–75.05

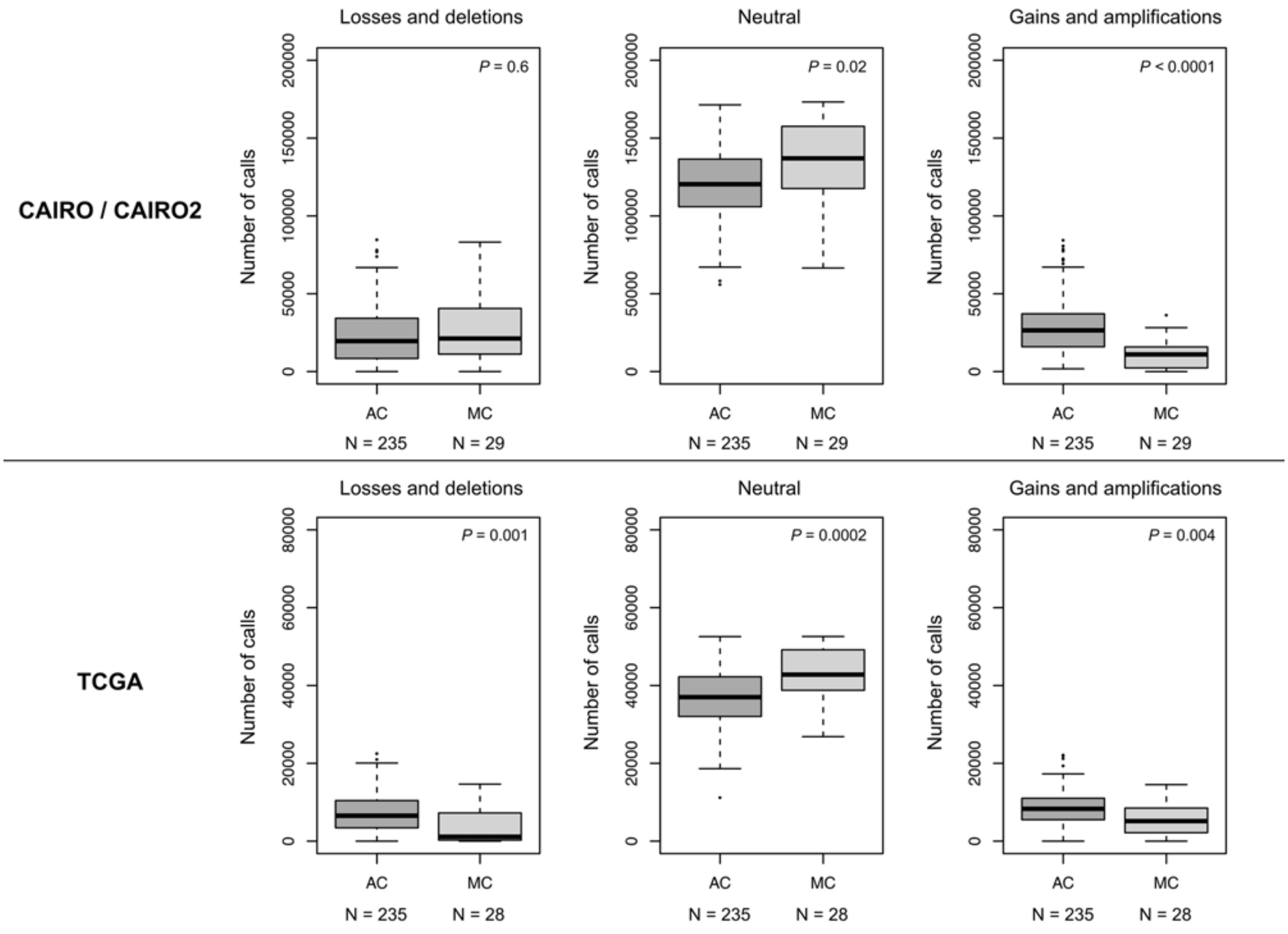

Supplementary Figure S1: Levels of chromosomal instability. The box plots show the number of calls per cohort that have been categorized as loss or deletion, neutral, or gain or amplification for the AC and MC samples. In the CAIRO/CAIRO2 cohort there were particularly differences in the overall frequencies of gains between AC and MC. In the TCGA cohort the overall frequencies of both gains and losses were lower in MC compared with AC.

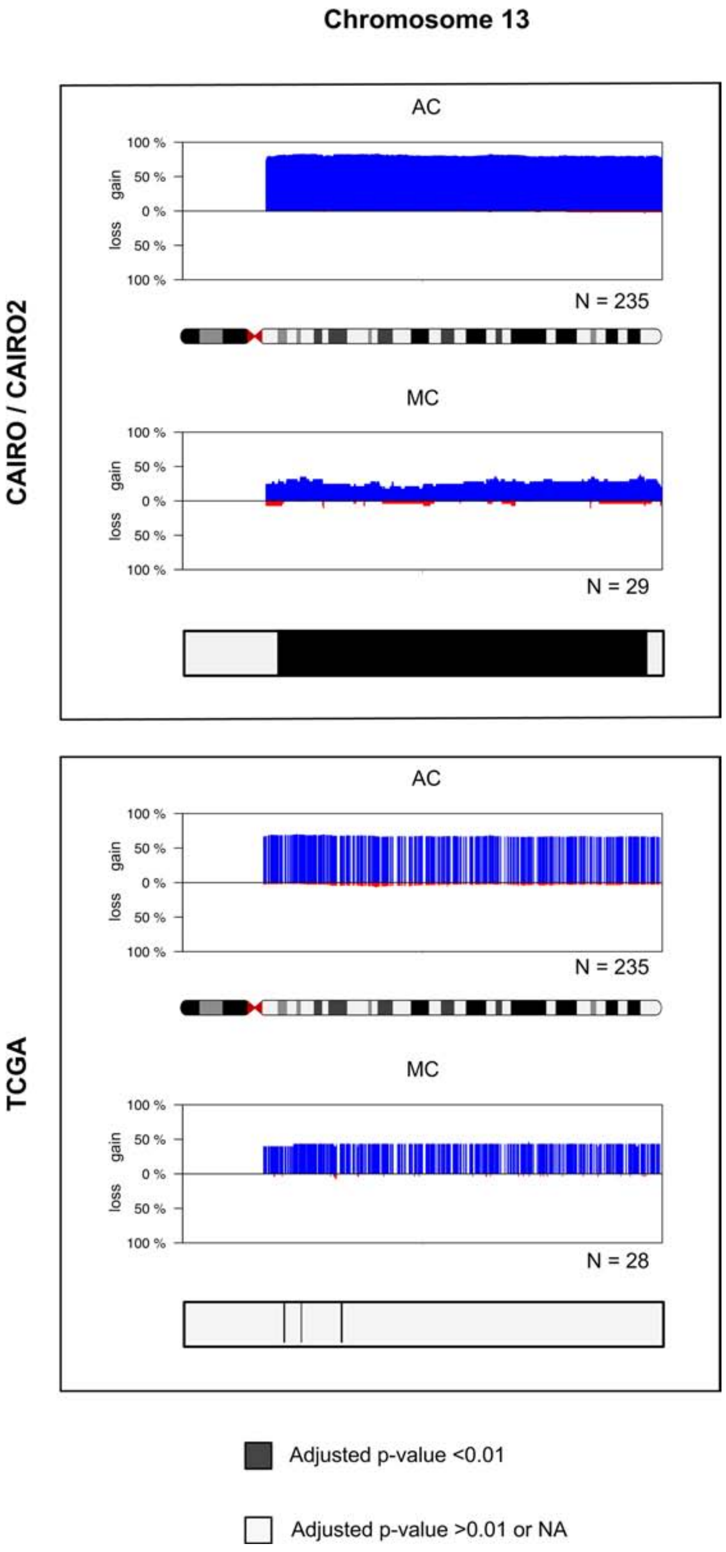

Supplementary Figure S2: Frequency plots of DNA CNAs in chromosome 13 determined in MC and AC patients from the CAIRO/CAIRO2 and TCGA cohorts. Probes on the array are ordered along the x-axis by their genomic position and the y-axis represents the frequency as the percentage of tumors with the respective gains (above zero; blue) or losses (below zero; red). The chromosomes represent ideograms with chromosomal bands. The corrected *p*-values obtained with statistical significance testing and correction for multiple testing are depicted in boxes below the plots. Black represents adjusted $P < 0.01$ and indicated a significant difference in DNA copy number between AC and MC; grey represents adjusted $P > 0.01$ and no indication of a significant difference.
